# Genomic regions involved in yield potential detected by genome-wide association analysis in Japanese high-yielding rice cultivars

**DOI:** 10.1186/1471-2164-15-346

**Published:** 2014-05-08

**Authors:** Jun-ichi Yonemaru, Ritsuko Mizobuchi, Hiroshi Kato, Toshio Yamamoto, Eiji Yamamoto, Kazuki Matsubara, Hideyuki Hirabayashi, Yoshinobu Takeuchi, Hiroshi Tsunematsu, Takuro Ishii, Hisatoshi Ohta, Hideo Maeda, Kaworu Ebana, Masahiro Yano

**Affiliations:** National Institute of Agrobiological Sciences, 2-1-2 Kannondai, Tsukuba, Ibaraki, 305-8602 Japan; NARO Institute of Crop Science, 2-1-18 Kannondai, Tsukuba, Ibaraki, 305-8518 Japan; NARO Institute of Vegetable and Tea Science, 360 Kusawa, Ano, Tsu, Mie, 514-2392 Japan; NARO Tohoku Agricultural Research Center, 3 Yotsusya, Daisen, Akita, 014-0102 Japan; NARO Hokuriku Agricultural Research Center, 1-2-1 Inada, Jyoetsu, Niigata, 943-0193 Japan

**Keywords:** Rice, High yield, *Indica*, *Japonica*, Introgression, Single-nucleotide polymorphisms (SNPs), Association mapping

## Abstract

**Background:**

High-yielding cultivars of rice (*Oryza sativa* L.) have been developed in Japan from crosses between overseas *indica* and domestic *japonica* cultivars. Recently, next-generation sequencing technology and high-throughput genotyping systems have shown many single-nucleotide polymorphisms (SNPs) that are proving useful for detailed analysis of genome composition. These SNPs can be used in genome-wide association studies to detect candidate genome regions associated with economically important traits. In this study, we used a custom SNP set to identify introgressed chromosomal regions in a set of high-yielding Japanese rice cultivars, and we performed an association study to identify genome regions associated with yield.

**Results:**

An informative set of 1152 SNPs was established by screening 14 high-yielding or primary ancestral cultivars for 5760 validated SNPs. Analysis of the population structure of high-yielding cultivars showed three genome types: *japonica-*type, *indica-*type and a mixture of the two. SNP allele frequencies showed several regions derived predominantly from one of the two parental genome types. Distinct regions skewed for the presence of parental alleles were observed on chromosomes 1, 2, 7, 8, 11 and 12 (*indica*) and on chromosomes 1, 2 and 6 (*japonica*). A possible relationship between these introgressed regions and six yield traits (blast susceptibility, heading date, length of unhusked seeds, number of panicles, surface area of unhusked seeds and 1000-grain weight) was detected in eight genome regions dominated by alleles of one parental origin. Two of these regions were near *Ghd7*, a heading date locus, and *Pi-ta*, a blast resistance locus. The allele types (i.e., *japonica* or *indica*) of significant SNPs coincided with those previously reported for candidate genes *Ghd7* and *Pi-ta*.

**Conclusions:**

Introgression breeding is an established strategy for the accumulation of QTLs and genes controlling high yield. Our custom SNP set is an effective tool for the identification of introgressed genome regions from a particular genetic background. This study demonstrates that changes in genome structure occurred during artificial selection for high yield, and provides information on several genomic regions associated with yield performance.

**Electronic supplementary material:**

The online version of this article (doi:10.1186/1471-2164-15-346) contains supplementary material, which is available to authorized users.

## Background

Rice (*Oryza sativa* L.) is a staple food in Asian countries. The population of Asian countries has almost tripled over the past 50 years and today accounts for 60% of the world population [1]. To supply the necessary rice for food and other uses, rice breeders continue to look for new ways to increase yield. In the 1960s, high-yielding semi-dwarf cultivars such as IR8 were first released [2]. Semi-dwarf cultivars were bred to be resistant to lodging under high nitrogen levels, which achieved their high yield [3]. IR8 was derived from crossing the Taiwanese semi-dwarf cultivar Dee Geo Woo Gen, which carries the *semi-dwarf 1* (*sd1*) gene, and the Indonesian cultivar Peta [2, 4]. The *sd1* gene derived from IR8 or other cultivars has played a crucial role in the breeding of high-yielding rice.

Increasing grain size or weight can improve yield by enlarging the sink size. Previous studies [5–10] have identified several genes associated with sink size and have shown that these genes could increase the 1000-grain weight of rice. The ability to produce fully mature seed is also an important trait for high-yielding rice because immature seeds reduce not only total grain weight, but also grain quality. Recently, allelic differences in the rice flowering-time gene *DTH2* were found to influence seed maturation [11]. Another gene controlling flowering time, *Ghd7*, has been reported to regulate traits involved in yield potential, such as plant height and the number of spikelets per panicle [12]. However, no single gene among those identified as controlling sink size can fully improve rice yield on its own. Therefore, the association between sink size genes and yield traits needs to be clarified by using a diverse genetic population.

Because of the utilization of rice for forage and bioethanol production in Japan, the materials used for breeding of high-yielding rice cultivars have changed drastically since the mid-1980s. Semi-dwarf *indica* cultivars have been extensively used as parental lines and crossed with Japanese *japonica* cultivars to produce high-yielding rice cultivars for Japan [13].

Thus, it is likely that the introgression of genomic regions from *indica* cultivars has contributed to the improvement of yield and other traits such as disease resistance in current Japanese high-yielding rice cultivars.

To identify the genes needed to produce high-yield rice cultivars, it is essential to develop genetic tools for the molecular dissection of current high-yielding cultivars and other breeding materials. Recently, next-generation sequencing technology has identified genome-wide single-nucleotide polymorphisms (SNPs) among diverse rice accessions. Large numbers of SNPs, which are often used in combination with high-throughput genotyping systems, have been widely used for genetic diversity analyses of diverse populations [14–19], breeding materials [20–22] and for mutation analyses [23]. Moreover, associations between traits and SNPs have been discovered by genome-wide association mapping in diverse populations [16, 19, 24]. These research platforms will allow us to elucidate genomic regions involved in yield potential and to accelerate the introgression of desirable genes into breeding materials.

In the present study, we used a genome-wide association study (GWAS) strategy to identify genomic regions contributing to high yield in Japanese rice cultivars derived from *indica–japonica* crosses. To dissect the fine genomic structures of admixed cultivars, we established a novel SNP set from previously discovered SNPs. We identified regions within the high-yielding cultivars with high frequencies of either *indica* or *japonica* alleles and detected associations of these regions with traits contributing to high yield in Japanese cultivars.

## Results

### Development of a SNP set for the analysis of genomic constitutions of Japanese high-yielding rice cultivars

**Figure 1:**
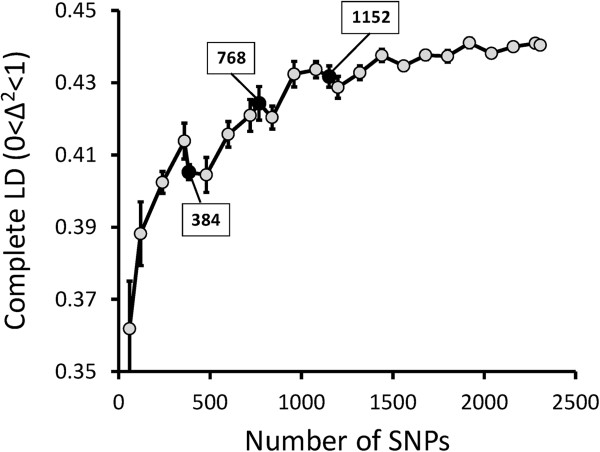
**Mean values of complete linkage disequilibrium (complete LD; 0 < Δ** ^**2**^ **< 1) estimated for SNP sets of different sizes.** SNP sets of different sizes were randomly chosen across the genome (10 times per set size), and mean values of complete LD were calculated for each set size. Vertical bars show the standard error.

A high-density SNP set is essential for detailed analysis of genome constitution. To obtain a set of informative SNPs evenly distributed across the rice genome, we surveyed 5760 SNPs in 14 high-yielding or primary ancestral cultivars (Akenohoshi, Akihikari, Hokuriku 193, Hoshiyutaka, Kanto PL12, Kochihibiki, Lemont, Milyang 23, Oochikara, Ooseto, Suweon 258, Tachisugata, Tainung 67, and Takanari; Additional file 1: Table S1; Additional file 2: Table S2). A subset of 2307 informative SNPs was selected on the basis of no missing data and the absence of low-frequency alleles (i.e., those found in fewer than 3 of the 14 cultivars). By performing a simulation for complete linkage disequilibrium (LD), which was estimated by comparing the genotypes of pairs of neighboring SNP alleles, we estimated the number of SNPs required for practical use in the analysis of genomic constitutions of Japanese high-yielding rice cultivars. As the number of SNPs increased, the mean value of complete LD also increased, but reached a plateau at approximately 1000 SNPs (Figure 1). Using this information and considering the analytical system and cost for the analysis, we randomly selected 1152 SNPs from among the 2307 informative SNPs as a practical number for this analysis (Additional file 3: Figure S1).

### Genome classification of Japanese high-yielding rice cultivars

The genetic diversity of Japanese high-yielding rice cultivars was estimated from the polymorphism information content (PIC) value and related values such as heterozygosity. These values were not strongly affected by sample size and were larger for samples of Japanese high-yielding rice cultivars (HY) than for samples of either domestic (PD) or overseas (PO) parents, which contained both *japonica* and *indica* cultivars (Table 1). They were also larger than those of the *indica* and *japonica* samples in both WRC and JRC populations (*seeMethods*).

**Table 1:** **Genetic features of 1046 SNPs screened from a subset of 1152 SNP markers for five rice populations**

					Adjusted sample size (n = 14)		
	Sample size	MAF	Heterozygosity	PIC	MAF	Heterozygosity	PIC
All	126	0.338	0.010	0.335			
HY	35	0.307	0.010	0.318	0.299	0.010	0.307
PO	14	0.264	0.010	0.279	0.264	0.010	0.279
PD	29	0.150	0.008	0.160	0.142	0.008	0.150
*Indica*	24	0.106	0.011	0.119	0.104	0.010	0.115
*Japonica*	24	0.198	0.013	0.213	0.196	0.013	0.210

MAF, minor allele frequency; PIC, polymorphism information content.

HY, high-yielding cultivar; PO, parental cultivar (overseas); PD, parental cultivar (domestic).

Because Japanese high-yielding rice cultivars were derived from crosses between *japonica* and *indica* cultivars (Additional file 4: Figure S2), the genomes of many high-yielding cultivars would be expected to represent an admixture of *indica* and *japonica* genome types. To show the extent of admixture, a structure analysis was applied to Japanese high-yielding cultivars and other reference cultivars with taxon information. For an assumed number of populations, structure analysis can estimate the proportional contribution of each ancestral population to each cultivar.

**Figure 2:**
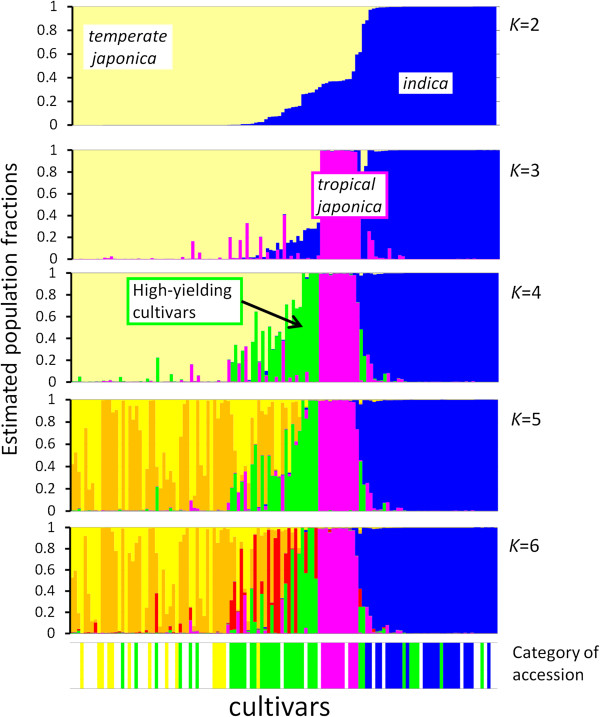
**Structure analysis of 126 rice cultivars using models of two to six ancestral groups.** In the upper five parts of the graph, each vertical bar represents a single cultivar; values displayed are the estimated population fractions in each cluster. Yellow (orange and red), blue, magenta and green indicate genome components from *temperate japonica*, *indica*, *tropical japonica* and high-yielding cultivars, respectively. The bottom panel of the figure indicates the category to which each accession belongs (see Additional file 5: Table S3). Colors have the same meaning as above. Accessions shown in white are overseas *indica* (PO-*indica*) or domestic (PD) parental cultivars.

The structure obtained for *K* (number of populations) = 3 seemed to correspond to *japonica*, *indica*, and an admixture of the two (Figure 2). At *K* = 4, the admixture group was subdivided into two groups, one representing an admixture of *indica* and *japonica* cultivars, the other containing *tropical japonica* cultivars*.* These structures were compared to the categories of accessions determined by previous studies (Additional file 5: Table S3). The structure consisting of four groups (*K* = 4) best fit the clustering results.

### Combination of genomic regions derived from overseas and domestic parental cultivars in genomes of Japanese high-yielding rice

**Figure 3:**
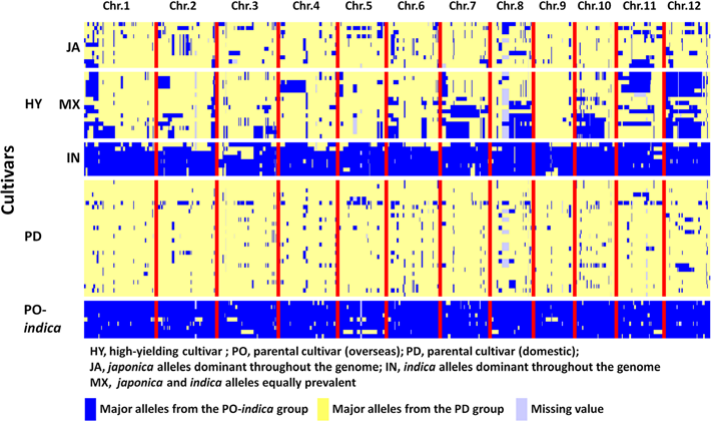
**Graphical genotypes of Japanese high-yielding (HY), overseas indica (PO-** ***indica*** **) and domestic (PD) parental cultivars based on 649 SNPs chosen to discriminate alleles derived from PD and PO-** ***indica*** **cultivars.** Each row represents one cultivar. Rows corresponding to HY cultivars are arranged in the same order (right to left) as in Additional file 6: Figure S3. The rows corresponding to PD cultivars are arranged in order of cultivar number (Additional file 1: Table S1). The 649 SNPs were chosen as having a difference of major allele frequency between the PD and PO-*indica* groups of >0.7.

Structure and cluster analysis showed that most of the overseas parental cultivars were classified into the *indica* group and the domestic parents into the *japonica* group (Figure 2; Additional file 6: Figure S3). To determine the genome type most prevalent in each chromosomal region, we focused on 649 of the 1152 SNPs. This subset was able to discriminate between the overseas *indica* (PO-*indica*) and domestic *japonica* (PD) parental materials and allowed us to determine the distribution of *japonica* and *indica* alleles in the genome of Japanese high-yielding rice cultivars (Figure 3). The Japanese high-yielding cultivars showed different levels of genome-type mixing and could be generally classified into three types: *japonica* alleles dominant throughout the genome (type JA), *indica* alleles dominant throughout the genome (type IN) and an even mixture of both types (type MX). These three types corresponded to the *japonica* type, *indica* type and admixture type classified in the structure and cluster analyses. Although the high-yielding cultivars differed widely in genome structure, in certain chromosome regions of the mixtures, one genome type or the other seemed to dominate; for example, the short arm of chromosome 1 and most of chromosomes 11 and 12 contained predominantly *indica* alleles (Figure 3).

**Figure 4:**
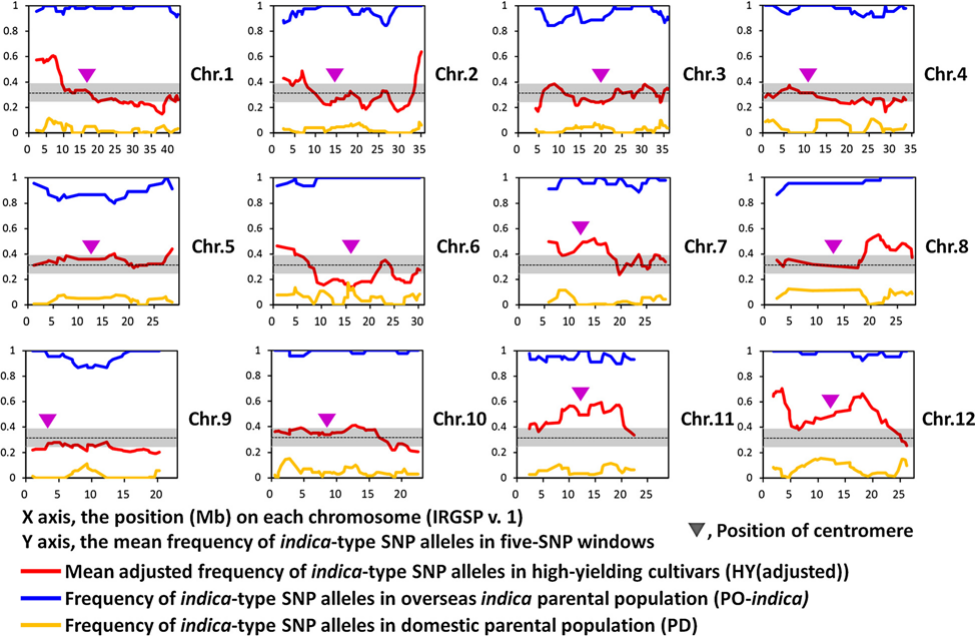
**SNP allele frequency of** ***indica*** **type in a Japanese high-yielding rice population and parental cultivars.** To reduce bias caused by parental allele frequency, HY allele frequencies were adjusted as described in Methods. Black dashed line and gray shading indicate the median and range (25th–75th percentile) of all mean values of HY (adjusted).

We calculated the allele frequency of *indica*-type alleles at each SNP in Japanese high-yielding, domestic and *indica* parental cultivars (Figure 4). Distinct regions dominated by the *indica* genome type were observed on the short arm of chromosome 1, the end of the long arm of chromosome 2, the middle of chromosome 7, the long arm of chromosome 8 and all of chromosomes 11 and 12. On the other hand, some regions were dominated by alleles from domestic parents, i.e., the long arm of chromosome 1, most of the long arm of chromosome 2 and the middle of chromosome 6.

### Phenotype annotation for frequently introgressed regions of *indica* or *japonica* genomes

We hypothesized that genomic regions of Japanese high-yielding cultivars that were dominated by a particular genome type (i.e., *japonica* or *indica*) might be involved in yield-related traits. To test this hypothesis, we performed association mapping in a population consisting of 68 selected lines with different yield-related traits and then examined the identified regions for highly skewed allele frequencies of either the *indica* or the *japonica* type. Significant associations (with permutation *P* < 0.01) were detected at eight SNP loci for six yield-associated traits (Table 2; Additional file 7: Figure S4). Associations with surface area of unhusked seeds were detected at four loci and associations with five other traits (blast susceptibility, heading date, number of panicles, 1000-grain weight and length of unhusked seeds) were detected at one or two loci each. All of these loci were located in genomic regions dominated by *indica*-type alleles (mean allele frequencies of 0.41–0.62) except for one locus at 21.84 Mb on chromosome 10, which was dominated by the *japonica*-type allele. A locus at 34.80 Mb on chromosome 2 showed correlation with surface area of unhusked seeds, length of unhusked seeds and 1000-grain weight. Interestingly, the values of all traits were smaller with *indica*-type alleles than with *japonica*-type alleles at this locus. A significant association was observed between surface area of unhusked seeds and loci at both 10.15 and 11.63 Mb on chromosome 7 and at 19.61 Mb on chromosome 8. At each of the three loci*,* the *indica* allele increased the surface area. At 10.15 Mb on chromosome 7, the *indica* allele was significantly associated with earlier heading. At 21.84 Mb on chromosome 10, the *indica* allele was associated with significantly reduced unhusked seed length. At 8.38 Mb on chromosome 11, the *indica* allele was associated with reduced panicle number. At 9.10 and 21.22 Mb on chromosome 12, the *indica* allele was associated with increased blast resistance.

**Table 2:** **SNPs associated with yield-related traits**

							Candidate gene			Allele effect		QTL			
Marker^a^	Chr	Position (Mb)	Traits	Mean allele frequency of HY	–log10(***P*** )	Permutation ***P***	Name	OsID	Position (Mb)	PO-indica	PD	QTL-ID^b^	Name	LOD	Interval or co-segregating marker
NIAS_Os_aa02003989			Surface area of unhusked seeds		5.12	0.001	**-**	**-**	**-**	19.68 ± 1.57	23.15 ± 3.26	108	gw2.1	3.25	RM250–RM208
	2	34.80	Length of unhusked seeds	0.62	4.67	0.003	**-**	**-**	**-**	8.26 ± 0.52	8.79 ± 0.76	362	**-**	3.99	C747–C1145
			1000-grain weight		5.48	0.003	**-**	**-**	**-**	22.54 ± 2.80	28.78 ± 4.94	**-**	**-**	**-**	**-**
NIAS_Os_ac07000274			Heading date		5.52	0.001				115.10 ± 7.62	128.67 ± 7.14	**-**	**-**	**-**	**-**
	7	10.15		0.41			*Ghd7*	Os07g 0261200	9.15						
			Surface area of unhusked seeds		5.67	0.001				20.57 ± 2.27	18.05 ± 0.92	**-**	**-**	**-**	**-**
NIAS_Os_ab07000535	7	11.63	Surface area of unhusked seeds	0.47	4.83	0.003	**-**	**-**	**-**	20.53 ± 2.27	20.53 ± 2.27	**-**	**-**	**-**	**-**
NIAS_Os_ah08001148	8	19.61	Surface area of unhusked seeds	0.51	5.00	0.001	**-**	**-**	**-**	20.55 ± 2.26	19.37 ± 2.27	**-**	**-**	**-**	**-**
NIAS_Os_aa10003574	10	21.84	Length of unhusked seeds	0.21	4.52	0.004	**-**	**-**	**-**	8.26 ± 0.52	8.51 ± 0.70	211	**-**	7.98	RZ811–RG561
P0943	11	8.38	Number of panicles	0.46	4.90	0.004	**-**	**-**	**-**	218.67 ± 30.74	287.55 ± 45.63	**-**	**-**	**-**	**-**
NIAS_Os_aa12004348	12	9.10	Blast susceptibility	0.44	5.22	0.009	*Pi–ta*	Os12g 0281300	10.61	0.17 ± 0.84	2.35 ± 2.13	**-**	**-**	**-**	**-**
P0926	12	21.22	Blast susceptibility	0.48	5.55	0.002	**-**	**-**	**-**	0.07 ± 0.45	2.26 ± 2.18	383	*Pi32(t)*	8.65	C449

^a^SNPs identified as having a significant permutation *P* -value (*P* < 0.01) detected by genome-wide association mapping in a high-yielding rice population. The SNPs chosen for testing represent frequently introgressed regions from *japonica* or *indica* parental cultivars.

^b^QTL ID from Q-TARO database.

By searching databases of functionally characterized genes and quantitative trait loci (QTL) in rice [25, 26], we identified two genes and four QTLs as candidates in these eight regions. *Ghd7*, which regulates flowering time and yield potential [12], was near the SNP at 10.15 Mb on chromosome 7, and the blast resistance gene *Pi-ta*[27] was near the SNP locus at 9.10 Mb on chromosome 12. Information in the QTL database Q-TARO [26] suggested that four QTLs for the traits related to yield or blast resistance (candidate QTLs) were located close to three of the SNP loci (34.80 Mb on chromosome 2 [2 QTLs], 21.84 Mb on chromosome 10 and 21.22 Mb on chromosome 12).

### Classification of *Ghd7* and *Pi-ta* alleles

To determine whether the allele types of candidate genes *Ghd7* and *Pi-ta* were consistent with those of nearby SNPs having significant phenotype associations, we surveyed the relevant genome sequences of five Japanese high-yielding cultivars and one overseas parental cultivar. *Indica-* or *japonica-*specific alleles have been reported in previous studies of both *Ghd7*[12, 28] and *Pi-ta*[27]. We then compared the allele types of the SNPs and candidate genes in the six cultivars. In *Ghd7*, the promoter region SNP S_555 and the predicted amino acids at four positions (122, 136, 174 and 233) in the five Japanese high-yielding cultivars coincided with the allele type at the nearest significant SNP locus, NIAS_Os_ac07000274 (Table 3). Four of the five high-yielding cultivars (all except Tachiaoba [HY30]) had an *indica*-type allele for the SNP locus and at the five positions within *Ghd7*, as did overseas parental cultivar Suweon 258. For *Pi-ta*, the predicted amino acid sequences at three positions (148, 158 and 176) corresponded to the same allele type as the nearest significant SNP, NIAS_Os_aa12004348. Thus, the SNP alleles nearest *Ghd7* and *Pi-ta* can be used as markers to discriminate the allele type at each of these two loci.

**Table 3:** **Comparison of SNP and candidate gene allele types in regions associated with significant trait differences**

Chr	Gene/marker	Position	Position	Mizuhochikara	Takanari	Hokuriku 193	Tachisugata	Tachiaoba	Suweon 258
		(IRGSP v. 1) Mb	(aa)	HY22	HY34	HY9	HY32	HY30	PO11
7	*Ghd7* promoter (S_555)^a^	9.15		**T**	**T**	**T**	**T**	C	**T**
7	*Ghd7* ^b^	9.15	122	**G** ^**c**^	**G**	**G**	**G**	E	**G**
7	*Ghd7* ^b^	9.15	136	**S**	**S**	**S**	**S**	G	**S**
7	*Ghd7* ^b^	9.15	174	**V**	**V**	**V**	**V**	D	**V**
7	*Ghd7* ^b^	9.15	233	**A**	**A**	**A**	**A**	P	**A**
7	NIAS_Os_ac07000274	10.15		**G**	**G**	**G**	**G**	T	**G**
7	NIAS_Os_ab07000535	11.63		**C**	**C**	**C**	**C**	**C**	**C**
12	NIAS_Os_aa12004348	9.10		**G**	**G**	**G**	T	T	**G**
12	*Pi-ta* ^d^	10.61	148	**R**	**R**	**R**	S	S	**R**
12	*Pi-ta* ^d^	10.61	158	**H**	**H**	**H**	Q	Q	**H**
12	*Pi-ta* ^d^	10.61	176	**D**	**D**	**D**	V	V	**D**

^a^SNP for discrimination of *Ghd7* haplotype (Lu *et al.* 2012) [28].

^b^Amino acid for discrimination of *Ghd7* allele (Xue *et al.* 2008) [12].

^c^Bold characters correspond to allele classified as *indica* type.

^d^Amino acid for discrimination of *Pi-ta* allele (Bryan *et al.* 2000) [27].

## Discussion

Yield is a complicated trait involving multiple component traits such as seedling vigor, photosynthetic rate, heading date and others; in turn, these traits are considered to be controlled by multiple genes. Therefore, it has been difficult to identify any single factor associated with increased yield potential in rice. Combining diverse alleles from *indica* and *japonica* is one way to produce desirable genotypes for high-yielding rice. To achieve this, cross-breeding has been conducted for over 30 years and, in fact, significant increases in yield potential have been achieved [13, 29]. It is expected that such high-yielding cultivars resulted from unique combinations of the *indica* and *japonica* genomes, but until now, no report has presented the genome-wide genotypes of such high-yielding cultivars. In this study, we selected informative SNP sets to visualize the whole-genome genotypes of such cultivars and identified various combinations of the *indica* and *japonica* genomes. Interestingly, the Japanese high-yielding rice cultivars could be divided into three groups: one dominated by *japonica* genome regions, one dominated by *indica* genome regions, and one containing admixtures. The combination of *indica* and *japonica* factors appears to have the greater potential for increasing yield because the admixture-type cultivars were most prevalent. Some chromosomal regions had highly skewed allele frequencies, suggesting that regions associated with high-yielding phenotypes had been conserved during the process of breeding selection.

GWAS is undoubtedly an effective way to detect QTLs, but is not very useful for a population consisting of improved cultivars, owing to the population structure. When using a population with a strong structure, the probability of detecting false-positive associations is higher than in a genetically divergent population. In spite of this risk, identification of valuable haplotypes underlying rice breeding populations is necessary to enable acceleration of the selection process. Here, we used the GWAS strategy to associate genomic regions having highly skewed *indica* or *japonica* allele frequencies with yield-related phenotypes. Several previous GWASs [15, 16, 19, 24] have shown that chromosomal regions associated with several characters, from simple domestication traits such as a glutinous phenotype to more complex traits such as flowering time and seed size, were introgressed from one cultivar group into another. In this study, we detected eight genome regions with significant phenotypic associations, two of which were near previously reported functionally characterized genes. Both the positions and the phenotypes associated with the latter two genome regions coincided with the positions and phenotypes of *Ghd7* and *Pi-ta*.

Heading date is an important trait that affects yield and plant type (i.e., harvest index). We detected a significant signal associated with heading date at position 10.15 Mb on chromosome 7, near *Ghd7*[12], and the type of SNP allele in each of six cultivars coincided with the type of *Ghd7* allele in that cultivar. The *indica* and *japonica* alleles found in Japanese high-yielding rice cultivars correspond to *Ghd7-1* and *Ghd7-2*, respectively. A previous study [12] concluded that *Ghd7-1* is a fully functional allele that confers late heading and that *Ghd7-2* is a weak functional allele that confers an intermediate phenotype. However, the allele effects of *Ghd7* in our materials were the opposite of those previously reported: strains containing the *Ghd7-1* (*indica*) allele flowered earlier than those containing *Ghd7-2* (Table 2, Additional file 8: Figure S5). Other studies have also found large variations in heading date among cultivars with the same *Ghd7* allele [12, 28]. These differences might be caused by the interaction of *Ghd7* with other genes controlling heading date.

Under the current system of rice cultivation, which uses paddy fields as efficiently as possible, cultivars with short growth duration from seeding to maturing may be suitable for obtaining optimal yield [30]. We also observed a negative correlation between heading date and surface area of unhusked seed (Additional file 8: Figure S5, Additional file 9: Table S4). The strong positive relationship between surface area of unhusked seed and 1000-grain weight might indicate that early heading contributes to an increase in seed weight per grain (Additional file 9: Table S4). Thus, we conclude that early heading associated with the *indica-Ghd7* allele is important for grain filling in Japanese high-yielding rice.

*Pi-ta* is one of several rice blast resistance genes, and the *indica*-type allele has been reported to confer resistance [27]. The *indica*-type allele found in Japanese high-yielding rice cultivars was identical to the corresponding resistance alleles of Yashiromochi (*Pi-ta*) and Tetep (*Pi-ta2*). It has also been suggested that progeny of Suweon 258 contain *Pi-ta* or *Pi-ta2*[13]. We conclude that the frequently introgressed region from *indica* rice detected at position 9.10 Mb of chromosome 12 contains an allele of *Pi-ta* that confers resistance.

Our analysis showed a second SNP (P0926) on chromosome 12 associated with blast susceptibility. Because it was located more than 10 Mb from SNP NIAS_Os_aa12004348 (near *Pi-ta*), other blast resistance genes might be located in this region. Studies of the distribution of disease resistance loci [31] and nucleotide-binding-site genes [32] in the rice genome have shown that many loci or genes associated with disease resistance are located in the middle part of chromosome 12. According to our data, parents from overseas were the donors of blast resistance genes now found in high-yielding rice in Japan.

In several other chromosomal regions that did not contain candidate genes, we detected significant SNPs co-located with four putative QTLs for yield traits. The reliability of the QTLs registered in Q-TARO [26] is indicated by LOD values, which were relatively high for these QTLs (3.25 to 8.65). Two putative QTLs [33, 34] near the end of the long arm of chromosome 2 were associated with grain weight, as was a SNP in this same region, NIAS_Os_aa02003989. Although this region was categorized as highly skewed towards *indica*-type alleles, the *japonica*-type allele was associated with higher grain weight. This direction of allele effect coincided with that of a previously identified QTL (QTL-ID# 362) [34]. Meanwhile, previous studies [33, 34] reported that QTLs for other yield traits were also co-localized in the same region. Notably, a QTL associated with grain number per panicle was detected in two previous QTL studies [33, 34], and the non-*japonica* allele identified in this region resulted in an increase in grain number per panicle [34]. In this genome region, the selection of QTLs for traits such as grain number per panicle might have been stronger than for grain size and weight.

A previously reported QTL [35] near the location of SNP NIAS_Os_aa10003574 on chromosome 10 was associated with 1000-grain weight. This SNP was associated with the length of unhusked seeds, which were longer when the *japonica*-type allele was present. The direction of allele effect was unclear in the previous study, but the candidate QTL was very reliable because it was detected in two different populations [35]. Therefore, it is possible that several QTLs for seed size might be located in this region.

Despite the association of yield-related phenotypes (surface area of unhusked seeds and number of panicles) with three other SNPs with highly skewed allele frequencies (Table 2), we could not find any candidate genes or QTLs in these regions. Characterization of currently unknown QTLs for yield such as these would contribute to the development of high-yielding rice.

We did not detect significant QTLs responsible for either of the direct yield traits (air-dry seed weight and air-dry total plant weight) examined in this study. This implies that the contribution of many QTLs with small effects and/or interaction among several QTLs, possibly with large effects, control these traits. However, our findings of the skewed allele frequencies in Japanese high-yielding rice cultivars and of significant QTLs controlling other yield-related phenotypes will help to elucidate the complicated genetic mechanisms controlling rice yield.

## Conclusions

Introgression breeding from *indica* to *japonica* or from *japonica* to *indica* is an established strategy for the accumulation of QTLs and genes controlling high yield while avoiding negative effects such as hybrid weakness, which is often a barrier to making wide crosses. Our informative SNP set is an effective tool for the identification of introgressed *indica* regions in *japonica* genetic backgrounds and vice versa. Additionally, we have demonstrated that phenotypic annotation of introgressed regions is possible. Future studies leading to additional phenotype annotations for introgressed genomic regions would accelerate the identification and accumulation of QTLs and genes for the development of high-yielding rice.

## Methods

### Plant materials and DNA extraction

A set of 35 Japanese high-yielding rice cultivars and 43 of their parental cultivars were subjected to SNP genotyping in this study (Additional file 1: Table S1). We used 14 core cultivars (Akenohoshi, Akihikari, Hokuriku 193, Hoshiyutaka, Kanto PL12, Kochihibiki, Lemont, Milyang 23, Oochikara, Ooseto, Suweon 258, Tachisugata, Tainung 67 and Takanari) to identify an appropriate SNP set for analysis of genetic architecture in Japanese high-yielding cultivars. To validate the SNP set, we added 31 accessions from the NIAS world rice core collection [36] and 17 accessions from the NIAS Japanese rice core collection [37] (Additional file 2: Table S2). Total DNA was extracted from young leaves of 10 plants from each cultivar by the CTAB method [38]. The total of 5760 SNPs were derived from three resources: comparisons between Nipponbare and Kasalath, Naba or Khau Mac Kho (unpublished data); comparisons between Nipponbare and Rikuu132 or Eiko [20]; and a comparison among a rice diversity research set [14]. SNP genotyping was carried out using the Illumina GoldenGate Bead Array technology platform (Illumina Inc., San Diego, CA, USA). For each sample, 250 ng of DNA was used. All experimental procedures for the SNP genotyping followed the manufacturer’s instructions.

To develop a SNP set for the analysis of genetic architecture of Japanese high-yielding cultivars, we estimated LD values as the pairwise Δ^2^ between neighboring SNPs for 24 sets containing different numbers of SNPs. The definition of Δ^2^ was equivalent to that in our previous study [22]. The mean complete LD (0 < Δ^2^ < 1) was estimated for different numbers of SNPs randomly chosen across the genome (10 times per set size). Finally, we selected a SNP set consisting of 1152 SNPs based on the mean value of complete LD. The 1152 SNPs are listed in Additional file 10: Table S5.

### Genetic diversity, structure and phylogenic analysis of Japanese high-yielding rice cultivars

We genotyped 126 rice cultivars (Additional file 5: Table S3) using the set of 1152 SNPs. The following criteria for the classification of a non-informative SNP were adopted: (1) no information on its genome position (IRGSP v. 1 [39, 40]), (2) heterozygosity or no signals detected in more than 5% of the accessions, and (3) an allele frequency of ≤2% (to minimize the risk of genotyping error). Using these criteria, we selected a total of 1046 informative SNPs and used them for the subsequent analysis.

Minor allele frequency, heterozygosity and PIC for five populations (subsets of the 126 cultivars) were calculated by analyzing the data obtained for the 1046 SNPs with PowerMarker v. 3.25 software [41]. To estimate the effect of sample size on these values, we recalculated each value by adjusting the sample size for each population to *n* = 14, corresponding to the sample size of the smallest population. The value for each statistic was the mean of 10 datasets, obtained by randomly picking 14 samples from the original population 10 times.

The population structure of Japanese high-yielding rice was analyzed using InStruct software [42] with the admixture model. To eliminate false-positive structures arising from excess SNPs, we selected 10 SNPs per chromosome that were nearly evenly distributed among each of chromosomes from the 1046 SNPs. The run-length parameters were 5000 burn-in iterations, and 100 000 replications per chain after the burn-in period using the Markov-chain Monte Carlo method. We used simulations with *K* values (number of populations in the model) ranging from 2 to 6, with five replications. Each structure (Figure 2, top five panels) was compared to the graph indicating the category of each accession determined by previous studies (Figure 2, bottom panel; Additional file 5: Table S3). A phylogenetic tree was constructed using the neighbor-joining method to analyze the 126 cultivars genotyped with 1046 SNP loci; analysis was implemented in the MEGA5 program [43]. The clusters were characterized by using reference cultivars belonging to two NIAS rice core collections (Additional file 5: Table S3).

### Estimation of allele frequencies differing between indica and japonica genome types in Japanese high-yielding rice cultivars

The presence of specific SNP alleles observed in the *indica* and *japonica* groups made it possible to discriminate alleles in high-yielding cultivars originating from either overseas *indica* (PO-*indica*, Additional file 1: Table S1) or domestic *japonica* (PD) parental cultivars. A genome type–specific allele was defined as one with a difference in allele frequency of greater than 0.7 between the two populations. To reduce bias due to parental allele frequency, an adjusted *indica* dominant allele frequency in Japanese high-yielding cultivars (HY) was calculated by each of allele frequency of PO-*indica* and PD. The calculation formula is written as adjusted HY = HY – PD – (1 – PO-*indica*). The median and range of 25th–75th percentiles of adjusted HY were calculated for five-SNP windows across the genome.

### Phenotype annotation of genomic regions with highly skewed indica or japonica allele frequencies

To associate phenotype data with regions having skewed frequencies of *indica* or *japonica* alleles, a test population segregating for these regions is essential. Therefore, we used 68 high-yielding breeding lines developed at the Institute of Crop Science, National Agriculture and Food Research Organization (NICS-NARO). These lines and the mean values for 14 yield-related traits are shown in Additional file 11: Table S6. Ten of the 14 traits (all except for 3 seed-related traits) were evaluated at NICS-NARO in 2009 with two replicates. Two traits related to disease susceptibility (blast and bacterial blast) were evaluated by an inoculation test with no replicates. Seed-related traits were measured by using the Smart Grain program [44]. Phenotype was annotated by the mixed linear model implemented in the program TASSEL v. 3.0 [45] with 290 SNP loci. The positions of SNP loci having both permutation *P* < 0.01 for a given trait and outside the range of 25th–75th percentiles of adjusted HY throughout the genome were used to search for candidate genes and QTLs for that trait. To look for functionally characterized genes that might explain an observed SNP (genome type) allele effect, we searched the OGRO database [25] for candidate genes in the same trait category and categorized as “natural variation” within a 4-Mb region centered on the SNP of interest. If no genes were found in this region, we searched the Q-TARO database [26] for QTLs harboring the relevant SNP or within 4 Mb of it.

### Classification of alleles of candidate genes by next-generation sequencing

Genomic DNA from six cultivars (Hokuriku 193, Mizuhochikara, Suweon 258, Tachiaoba, Tachisugata and Takanari) was extracted by the CTAB method [38]. An Illumina HiSeq 2000 sequencer was used to generate 100-bp paired-end reads (three samples per lane). Reads were mapped to the Nipponbare IRGSP v. 1 reference genome with BWA software [46], sorted and indexed with SAMtools [47]. To improve the raw alignment binary forms of SAM (BAMs) for variant calling, we realigned indels and recalibrated base quality scores using GATK software [48]. Duplicates were identified using Picard (http://picard.sourceforge.net). Variants (SNPs and indels) were called on each sample individually with the SAMtools mpileup algorithm. The filtering threshold was set as a quality score of ≮20. Variants detected in candidate genes were compared among the sequences of Nipponbare and the six cultivars.

### Availability of supporting data

Phylogenetic tree shown in Figure S3 (Additional file 6) has been deposited in TreeBASE (http://purl.org/phylo/treebase/phylows/study/TB2:S15706). Nucleotide sequence data is available in the DDBJ Sequenced Read Archive under the accession numbers DRP002297.

## Electronic supplementary material

Additional file 1: Table S1: List of Japanese high-yielding and parental rice cultivars used in this study. (XLS 74 KB)

Additional file 2: Table S2: List of WRC (World Rice Collection of NIAS) and JRC (Japanese Rice Collection of NIAS) accessions used in this study. (XLS 60 KB)

Additional file 3: Figure S1: Chromosomal distribution of the 1152 SNPs selected for this study. Vertical bars represent chromosomes 1 to 12 (from left to right), and red horizontal bars indicate the locations of SNPs. (PPTX 68 KB)

Additional file 4: Figure S2: Pedigree of Japanese high-yielding rice cultivars. Pedigree extends from left to right. Blue, overseas parents; yellow, domestic parents; green, cultivars from the world rice core collection (NIAS); mixed-color, high-yielding rice cultivars used in this study. The labels next to some boxes represent the cultivar numbers used in Additional file 1: Table S1 and Additional file 2: Table S2. (PPTX 298 KB)

Additional file 5: Table S3: Cluster assignment for each cultivar. (XLS 82 KB)

Additional file 6: Figure S3: Figure S3 Phylogenetic tree of 126 rice accessions, constructed using the neighbor-joining method to analyze data for 1046 SNP markers. The range of *japonica, indica* and *tropical japonica* was estimated from reference cultivars belonging to the NIAS Japanese and world rice core collections. Red arrows indicate the admixture-type of Japanese high-yielding cultivars as defined from the structure analysis. Other categories shown here are described in Additional file 1: Table S1. Cultivars are listed in Additional file 1: Table S1 and Additional file 2: Table S2). (PPTX 157 KB)

Additional file 7: Figure S4: Manhattan plots of GWAS (MLM) for six significant traits in 68 selected lines. The *x* axis shows the relative position on chromosomes 1 to 12, arranged with the short arm of each chromosome to the left. The *y* axis shows − log (*P*-value) of markers. Dashed line shows permutation *P* = 0.01; thus, points above the line represent markers with significant effects. BLAST, blast susceptibility; HD, heading date; 1000GW, 1000-grain weight; SEED AREA, surface area of unhusked seed; SEED LENGTH, length of unhusked seed; PANICLE NO., number of panicles. (PPTX 1007 KB)

Additional file 8: Figure S5: Correlation between heading data and surface area of unhusked seed. The correlation coefficient (*r*) was calculated for data for 68 high-yielding rice strains (see Additional file 11: Table S6). ***P < 0.0001. (PPTX 43 KB)

Additional file 9: Table S4: Pearson's correlation coefficients among 12 yield-related traits. (XLS 56 KB)

Additional file 10: Table S5: Core set of 1152 SNPs used for analysis of high-yield cultivars derived from *indica* and *japonica* crosses. (XLS 608 KB)

Additional file 11: Table S6: Yield-related traits of 68 high-yielding breeding lines developed at NICS-NARO used for phenotypic annotation of genome regions with highly skewed *indica* or *japonica* allele frequencies. All trait values are means of two replicates except for those of the two disease susceptibility traits (blast and bacterial blast), which were unreplicated. (XLS 85 KB)
