# Supplementary figures and images for: Genomic regions involved in yield potential detected by genome-wide association analysis in Japanese high-yielding rice cultivars
Source: BMC Genomics. 2014 May 8;15(1):346. doi: 10.1186/1471-2164-15-346 (PMC4035073; doi:10.1186/1471-2164-15-346)

## Slide 1
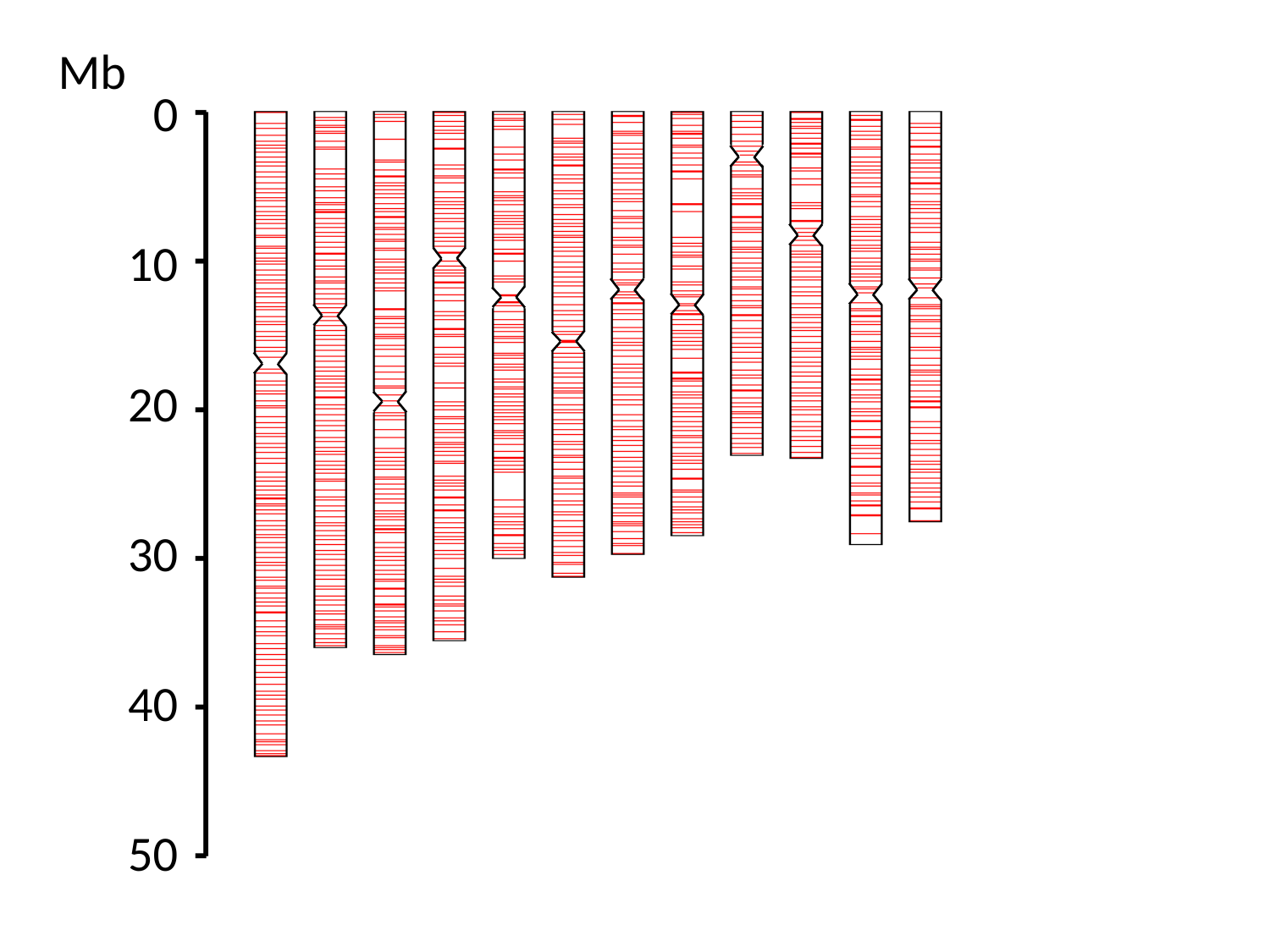

Mb
0
10
20
30
40
50

Supplement: Supplementary file 3 — Additional file 3: Figure S1: Chromosomal distribution of the 1152 SNPs selected for this study. Vertical bars represent chromosomes 1 to 12 (from left to right), and red horizontal bars indicate the locations of SNPs. (PPTX 68 KB) [file 12864_2013_6030_MOESM3_ESM.pptx]

## Slide 1
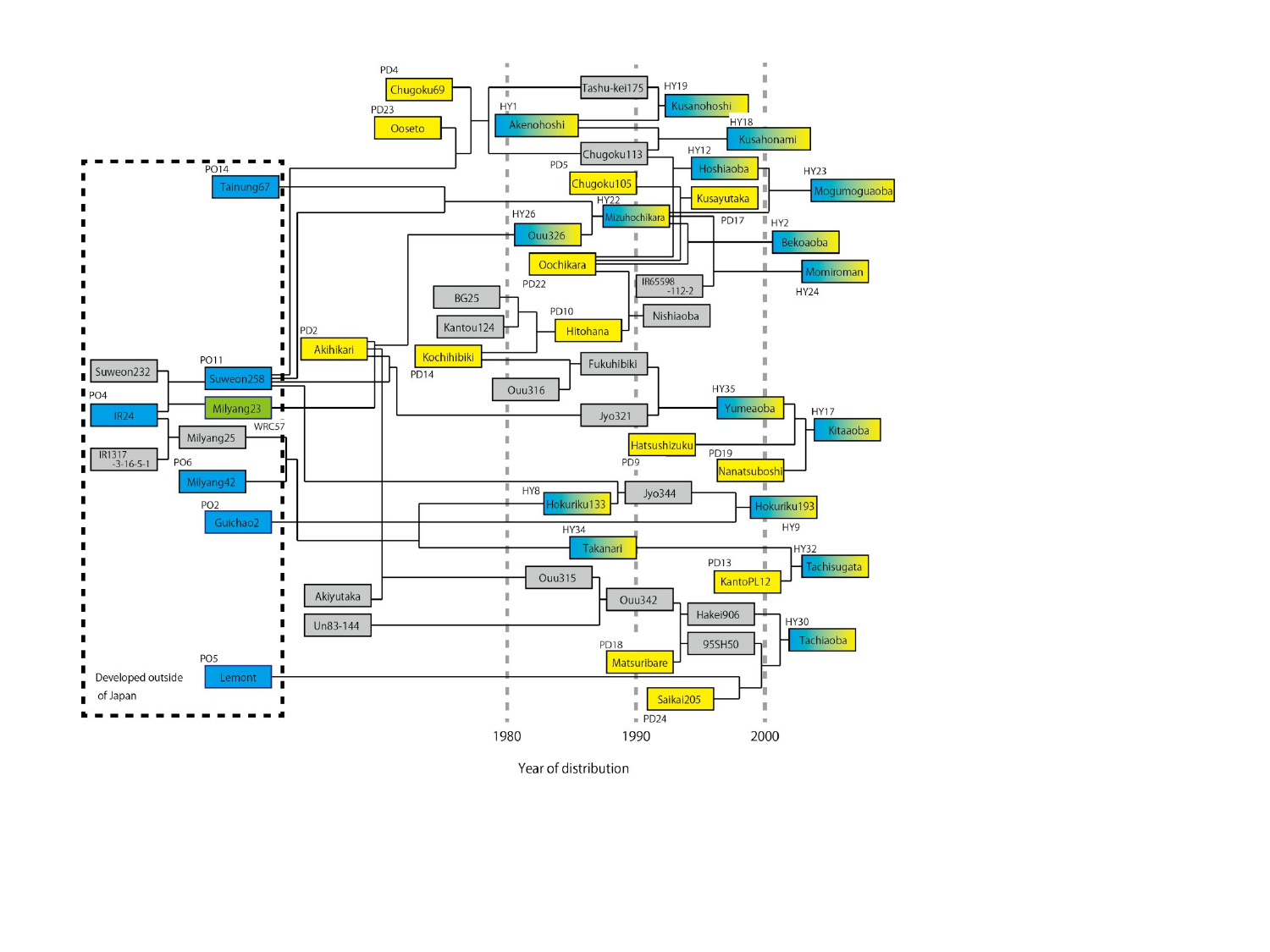

Supplement: Supplementary file 4 — Additional file 4: Figure S2: Pedigree of Japanese high-yielding rice cultivars. Pedigree extends from left to right. Blue, overseas parents; yellow, domestic parents; green, cultivars from the world rice core collection (NIAS); mixed-color, high-yielding rice cultivars used in this study. The labels next to some boxes represent the cultivar numbers used in Additional file 1: Table S1 and Additional file 2: Table S2. (PPTX 298 KB) [file 12864_2013_6030_MOESM4_ESM.pptx]

## Slide 1
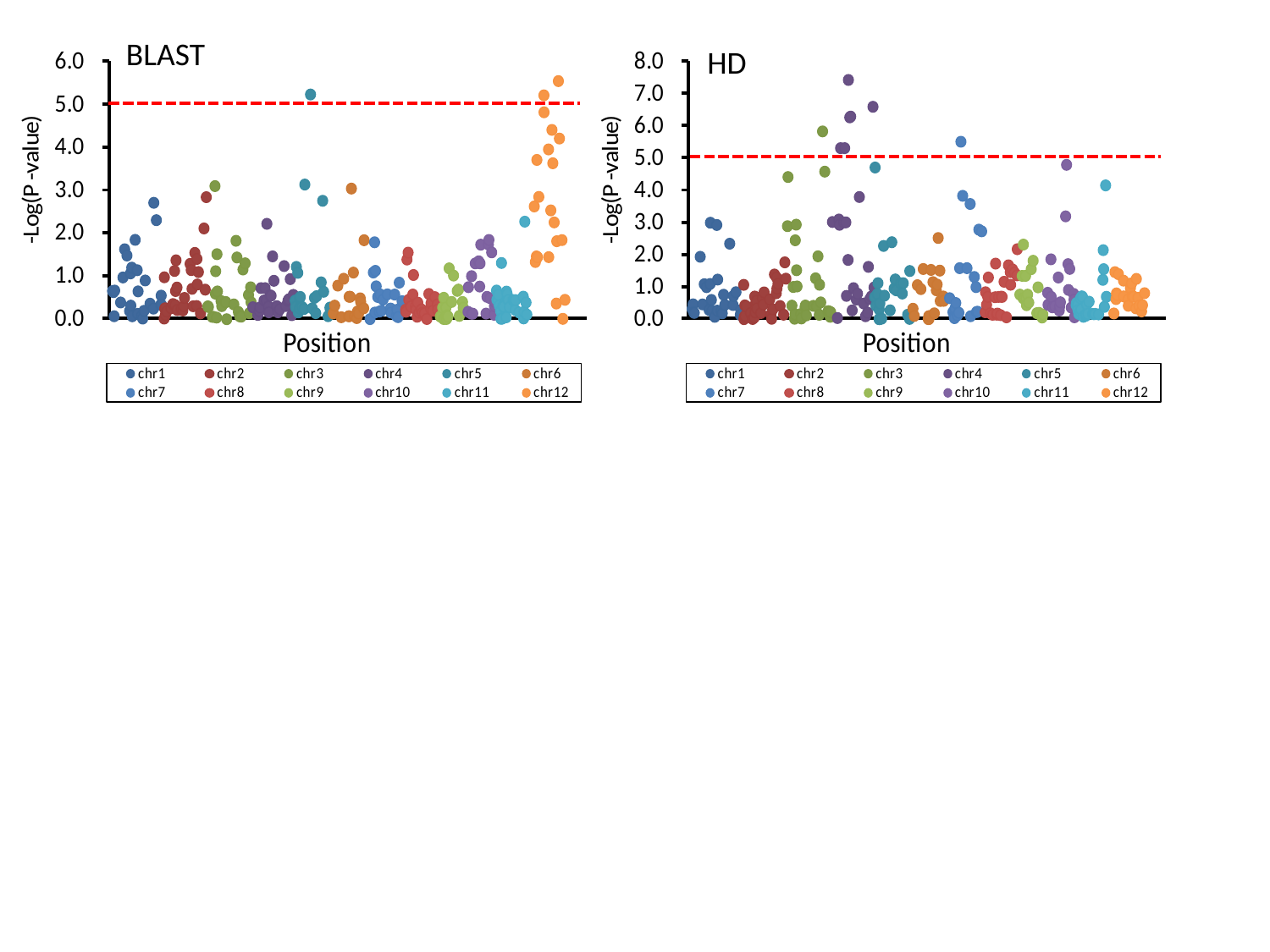

BLAST
HD

## Slide 2
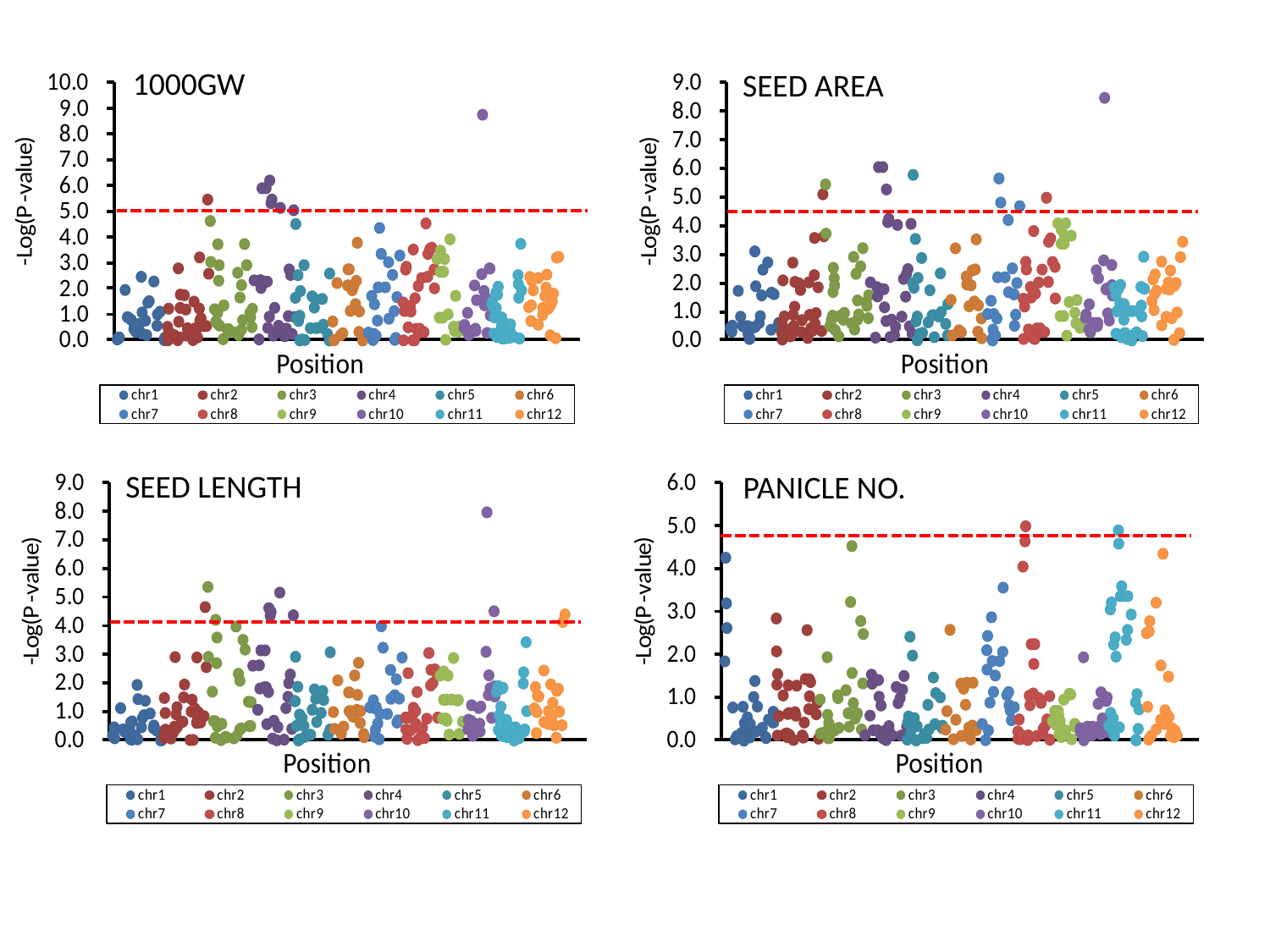

1000GW
SEED AREA
SEED LENGTH
PANICLE NO.

Supplement: Supplementary file 7 — Additional file 7: Figure S4: Manhattan plots of GWAS (MLM) for six significant traits in 68 selected lines. The x axis shows the relative position on chromosomes 1 to 12, arranged with the short arm of each chromosome to the left. The y axis shows − log (P-value) of markers. Dashed line shows permutation P = 0.01; thus, points above the line represent markers with significant effects. BLAST, blast susceptibility; HD, heading date; 1000GW, 1000-grain weight; SEED AREA, surface area of unhusked seed; SEED LENGTH, length of unhusked seed; PANICLE NO., number of panicles. (PPTX 1007 KB) [file 12864_2013_6030_MOESM7_ESM.pptx]

## Slide 1
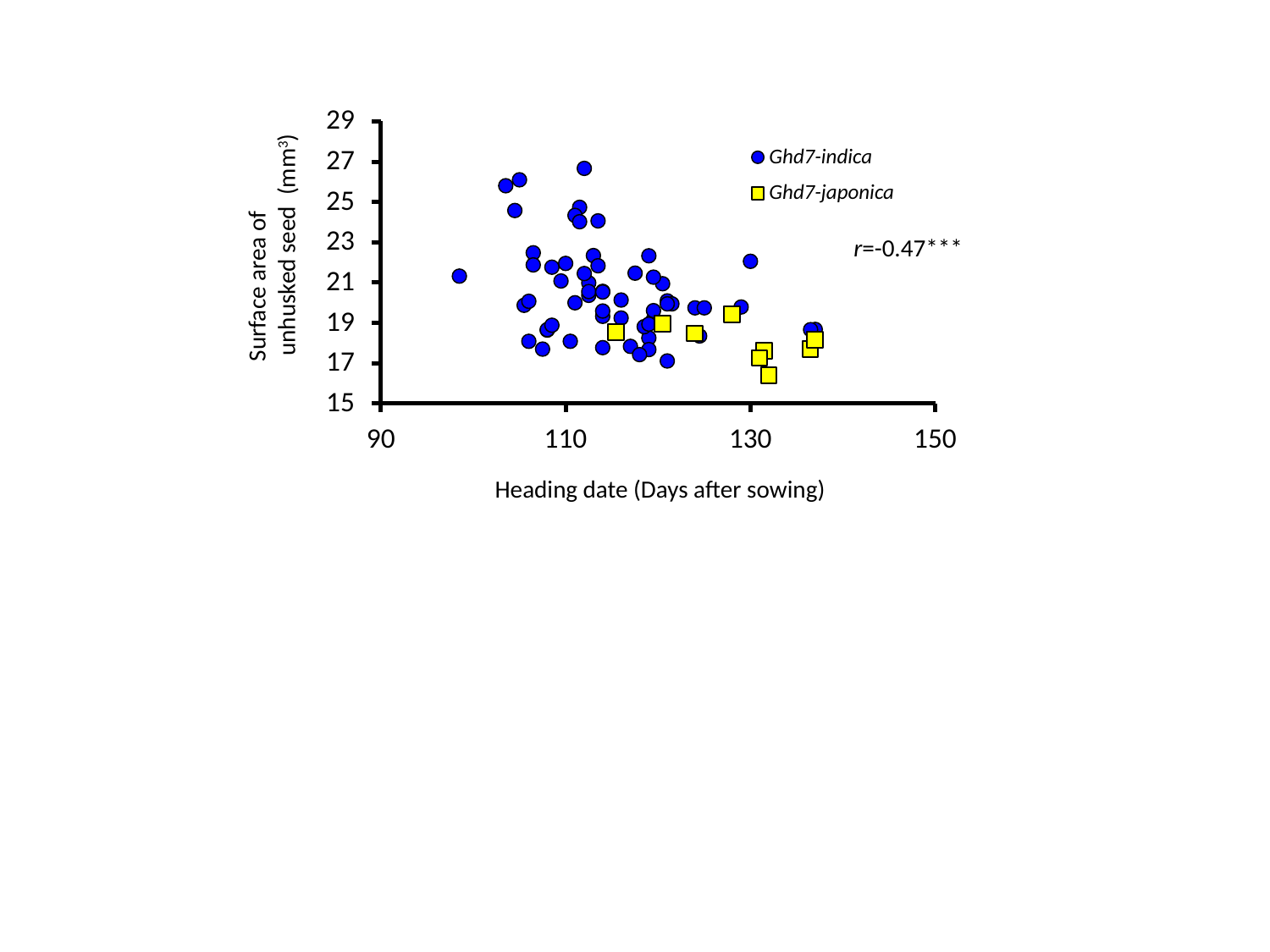

Surface area of
 unhusked seed (mm3)
r=-0.47***
Heading date (Days after sowing)

Supplement: Supplementary file 8 — Additional file 8: Figure S5: Correlation between heading data and surface area of unhusked seed. The correlation coefficient (r) was calculated for data for 68 high-yielding rice strains (see Additional file 11: Table S6). ***P < 0.0001. (PPTX 43 KB) [file 12864_2013_6030_MOESM8_ESM.pptx]
