# Supplementary material for: Genomic regions involved in yield potential detected by genome-wide association analysis in Japanese high-yielding rice cultivars
Source: BMC Genomics. 2014 May 8;15(1):346. doi: 10.1186/1471-2164-15-346 (PMC4035073; doi:10.1186/1471-2164-15-346)
Supplement: Supplementary file 6 — Additional file 6: Figure S3: Figure S3 Phylogenetic tree of 126 rice accessions, constructed using the neighbor-joining method to analyze data for 1046 SNP markers. The range of japonica, indica and tropical japonica was estimated from reference cultivars belonging to the NIAS Japanese and world rice core collections. Red arrows indicate the admixture-type of Japanese high-yielding cultivars as defined from the structure analysis. Other categories shown here are described in Additional file 1: Table S1. Cultivars are listed in Additional file 1: Table S1 and Additional file 2: Table S2). (PPTX 157 KB) [file 12864_2013_6030_MOESM6_ESM.pptx]

## Slide 1
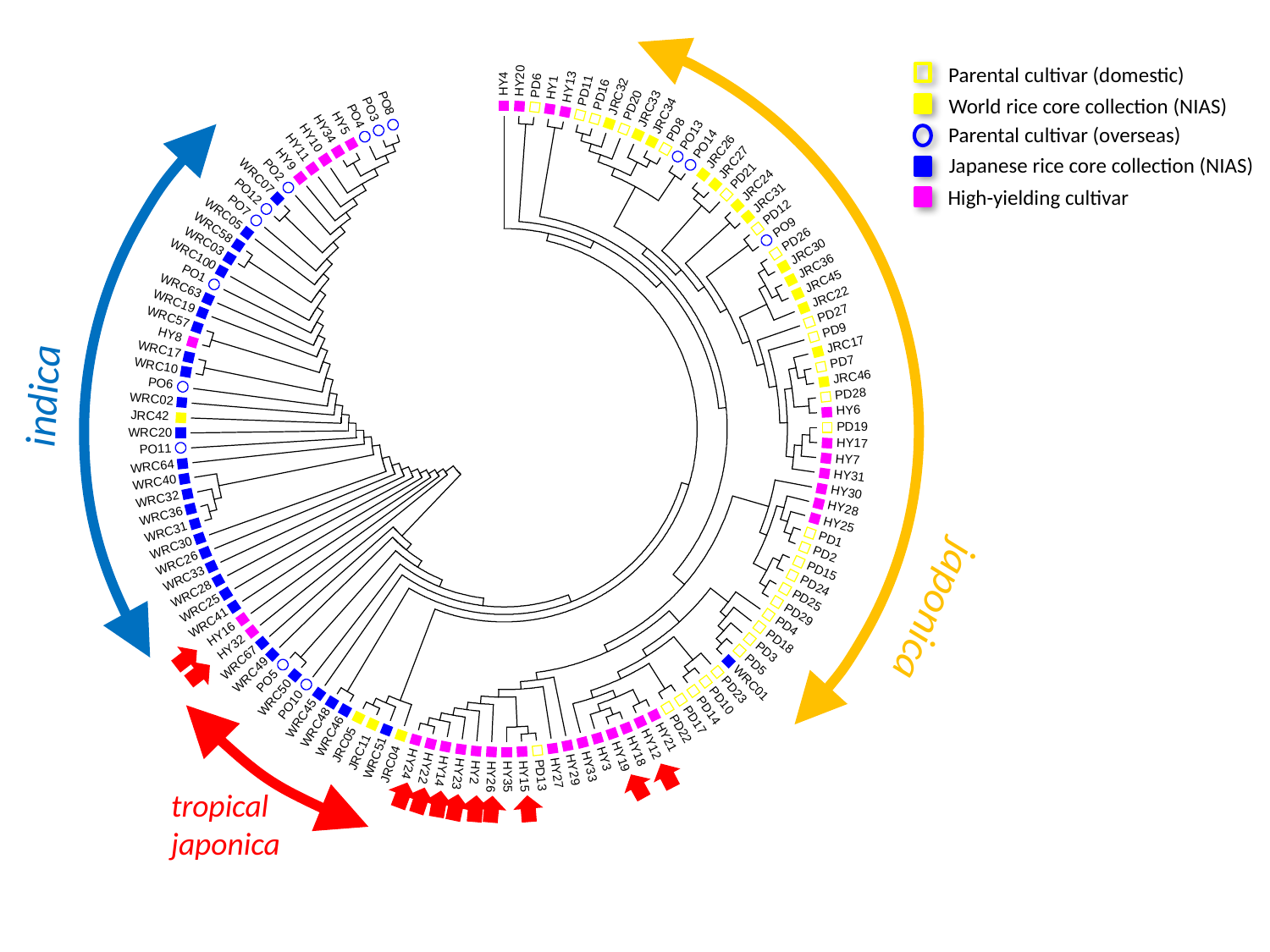

Parental cultivar (domestic)
World rice core collection (NIAS)
Parental cultivar (overseas)
Japanese rice core collection (NIAS)
High-yielding cultivar
indica
japonica
tropical
japonica
